# ABC/2 formula for “bedside” postoperative pneumocephalus volume measurement

**DOI:** 10.1186/s41016-022-00287-z

**Published:** 2022-08-03

**Authors:** David Yuen Chung Chan, Eric Yuk Hong Cheung, Ka Ho Hui, Cecelia Mei Sze Leung, Stephanie Chi Ping Ng, Wai Kit Mak, George Kwok Chu Wong, Tat Ming Danny Chan, Wai Sang Poon

**Affiliations:** Division of Neurosurgery, Department of Surgery, Faculty of Medicine, Prince of Wales Hospital, The Chinese University of Hong Kong, Hong Kong, SAR China

**Keywords:** ABC/2, Chronic subdural haematoma, Burr-hole, Pneumocephalus

## Abstract

**Background:**

Postoperative pneumocephalus is associated with a higher risk of recurrence of chronic subdural hematoma (cSDH). However, there is no verified simple way to measure the pneumocephalus volume at the bedside for daily clinical use. The ABC/2 method was shown to be a simple and reliable technique to estimate volumes of intracranial lesions, such as intracranial hematomas. This study aims to evaluate the accuracy of the ABC/2 formula in estimating volumes of pneumocephalus, as compared to the gold standard with computer-assisted volumetric analysis.

**Methods:**

A total of 141 postoperative computed tomographic (CT) brain scans of cSDH patients with burr-hole drainage were analysed. Pneumocephalus volume was measured independently by both the ABC/2 formula and the computer-assisted volumetric measurement. For the computer-assisted measurement, the volume of the air was semiautomatically segmented and calculated by computer software. Linear regression was used to determine the correlation between the ABC/2 method and computer-assisted measurement.

**Results:**

The postoperative pneumocephalus volume after bilateral burr-hole drainage was significantly larger than that of unilateral burr-hole drainage (29.34 ml versus 12.21 ml, *p* < 0.001). The estimated volumes by the formula ABC/2 significantly correlated to the volumes as measured by the computer-assisted volumetric technique, with *r* = 0.992 (*p* < 0.001). The Pearson correlation coefficient is very close to 1, which signifies a very strong positive correlation, and it is statistically significant.

**Conclusions:**

An excellent correlation is observed between the ABC/2 method and the computer-assisted measurement. This study verified that the ABC/2 method is an accurate and simple “bedside” technique to estimate pneumocephalus volume.

## Highlights

• The ABC/2 method is an accurate and simple “bedside” technique to estimate pneumocephalus volume.

• The postoperative pneumocephalus volume after bilateral burr-hole drainage was significantly larger than that of unilateral burr-hole drainage (29.34 ml versus 12.21 ml, *p* < 0.001).

• The estimated volumes of pneumocephalus by the formula ABC/2 were significantly correlated to the volumes as measured by the computer-assisted volumetric technique, with *r* = 0.992 (*p* < 0.001).

## Background

The presence of compressive pneumocephalus after burr-hole drainage has been reported to be associated with the recurrence of chronic subdural hematoma (cSDH) [1, 2]. However, mainly “thickness” and “midline shift” in centimetres (cm) were reported in these studies for pneumocephalus [3, 4]. You et al. in 2018 had reported that postoperative pneumocephalus volume greater than 30 ml had a significantly higher risk of cSDH recurrence [5]. In this study, pneumocephalus volume can be calculated by computed-volumetric measurement [5]. However, there are no verified simple “bedside” methods to measure volumes of pneumocephalus easily for daily clinical use. The ABC/2 method, or XYZ/2, is a reliable and simple technique to estimate volumes of intracranial pathologies such as intracranial hematomas [6, 7]. Using the ABC/2 formula, an excellent level of agreement between clinicians had been observed in estimating intracerebral haematomas (ICH) and epidural haematomas (EDH), acute subdural haematomas (aSDH), and chronic subdural haematoma (cSDH) volumes [8–13]. However, the accuracy of this formula for other intracranial conditions such as arteriovenous malformation (AVM) volume estimation was low [14]. It was reported that the ABC/2 formula overestimated cerebral AVM volume, and this has significant implications for stereotactic radiosurgery (SRS) planning [14]. The ABC/2 formula was also reported to be overestimating the volume of epidural hematoma (EDH) [15] and intracerebral haematoma (ICH) volume [16]. As a result, we cannot assume the same formula is applicable for pneumocephalus. To the best of our knowledge, there have not been studies specifically investigating the accuracy of this formula in measuring postoperative pneumocephalus volume in chronic subdural haematoma (cSDH) patients with burr-hole drainage.

The ‘bedside’ technique with ABC/2 can be a valuable tool in comparing volumes of postoperative intracranial air in cSDH patients after burr-hole drainage. This simple and reliable technique for pneumocephalus volume estimation can be of value for daily clinical application. It is also valuable to evaluate the treatment efficacy of various oxygen therapy for pneumocephalus in cSDH [17]. This study aims to evaluate the accuracy of the ABC/2 formula in estimating volumes of pneumocephalus, as compared to the gold standard with computer-assisted volumetric analysis.

## Methods

**Fig. 1:**
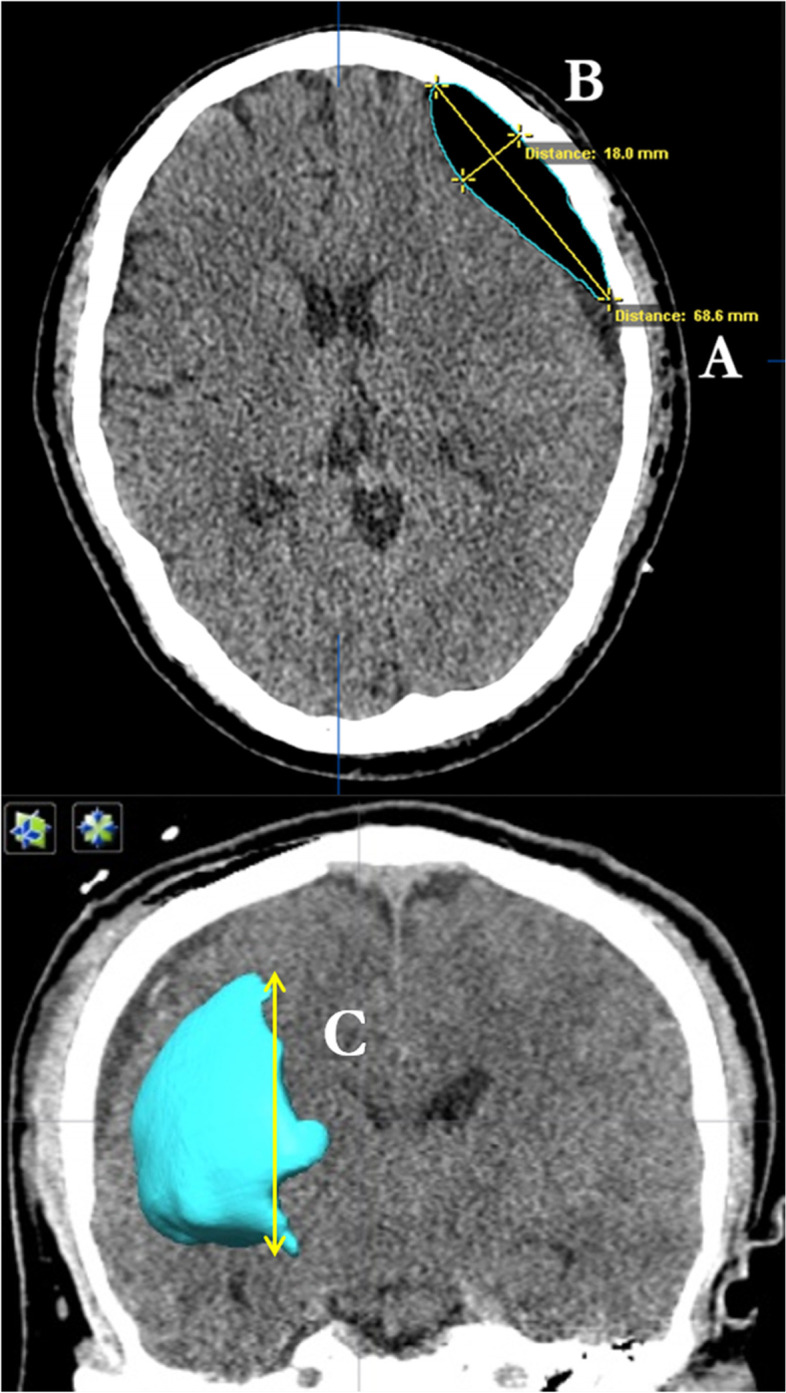
Axial postoperative CT brain scan of cSDH with pneumocephalus. For the ABC/2 method, “**A**” was defined as the largest longitudinal linear length of the pneumocephalus on the axial plane, “**B**” was the maximum width from the inner table of the skull to the cerebral cortex perpendicular to A on the same slice (upper), and “**C**” was the height of the pneumocephalus on a coronal plane, as determined by multiplying the number of CT scan slides with the pneumocephalus visible by the slice thickness (in this study, 5 millimetres (mm) in thickness). The estimated volume was a multiple of **A**, **B**, and **C** and then divided by 2. For the computer-assisted pneumocephalus volumetric measurement, semiautomatic segmentation was traced in each slice for computer-assisted volume calculation (lower) and 3D reconstruction of the pneumocephalus for visualization

We reviewed 141 postoperative plain CT brain scans of cSDH patients with burr-hole drainage performed during the period from 1st April 2020 to 31st December 2020 at the Prince of Wales Hospital, Hong Kong. Pneumocephalus volume was measured independently by both the ABC/2 formula and the computer-assisted volumetric measurement on the same set of plain CT brain scans. For the ABC/2 method, the first step is to select a representative slide near the centre of the pneumocephalus. “A” was defined as the largest longitudinal linear length in millimetre (mm) of the pneumocephalus on the axial plane, “B” was the maximum width (in mm) from the inner table of the skull to the cerebral cortex perpendicular to A on the same slice, and “C” was the height of the pneumocephalus on a coronal plane, as determined by multiplying the number of CT scan slides with the pneumocephalus visible by the slice thickness. The estimated volume was a multiple of A, B, and C and then divided by 2 (Fig. 1) For the computer-assisted measurement, Digital Imaging and Communications in Medicine (DICOM) data of the CT brain scans were analysed using the software Brainlab Elements (Brainlab Germany headquarters, Munich, Germany) and iPlan Cranial 3.0 software. The volume of the air was semiautomatically segmented and calculated by the software. A low attenuation value with −600 to −1000 Hounsfield units (HU) was set for the software to identify the intracranial air. The margin of the pneumocephalus was manually verified in each slice at 0.6-mm cut with auto-segmentation. The volume as calculated from the computer-assisted analysis was considered the gold standard for comparison. Linear regression was used to determine the correlation between the ABC/2 method and computer-assisted measurement.

Inclusion criteria were as follows (1) age greater than or equal to 18 years old; (2) radiological diagnosis of cSDH, as evidenced from CT brain scan; (3) admission under the care of the Division of Neurosurgery, Department of Surgery, Prince of Wales Hospital, Hong Kong; (4) treatment by burr-hole drainage; (5) cSDH patients with operations performed during the period from 1st April 2020 to 31st December 2020 at the Prince of Wales Hospital, Hong Kong SAR; and (6) postoperative CT brain scan performed within 96 h after the burr-hole operation. Exclusion criteria were as follows: (1) CT brain scans showing no or almost no pneumocephalus, (2) septations with multiloculated small intracranial air, and (3) volume of pneumocephalus less than 1 millilitre (ml), as measured using the computer-assisted volumetric analysis based on the DICOM. Postoperative CT brain scans of the patients fulfilling all the inclusion criteria and none of the exclusion criteria were analysed.

All participants in this study received the standard medical care at the Neurosurgery Unit. There were regular ward rounds by neurosurgeons to review the medical care at the neurosurgical wards.

Statistical analysis was performed with the Pearson correlation coefficient and linear regression. Statistical significance was set at 5%. Statistical analysis was performed with the Statistical Package for the Social Sciences for Microsoft Windows Version 25.0.0. (IBM SPSS Inc., Chicago, Illinois, USA).

Ethics approval was obtained from the Institutional Review Board of the Joint Chinese University of Hong Kong/Hospital Authority New Territories East Cluster (CUHK-NTEC CREC Ref. No.:2021.046).

All procedures performed in the studies involving human participants were in accordance with the ethical standards of the Institutional Research Committee and with the 1964 Helsinki Declaration and its later amendments or comparable ethical standards.

## Results

In total, 141 cSDH postoperative CT brain scans from 66 patients fulfilling the inclusion criteria and none of the exclusion criteria were analysed. Forty-five of the 66 patients had unilateral burr-hole drainage for cSDH, of which 19 were on the right side and 26 were on the left side. Twenty-one of the 66 patients had bilateral burr-hole drainage for cSDH. The mean age of patients was 71 years old (range: 41 years old to 98 years old). The mean pneumocephalus volume by the ABC/2 method was 16.26 ml (range: 0.20 to 123.40 ml) versus 18.04 ml (range: 1.11 to 128.45 ml) by computer-assisted measurement (Table 1). With a correlation coefficient of *r* = 0 being “no relationship” and *r* = 1 being a “perfect correlation,” the correlation by ABC/2 is excellent, with a correlation coefficient of *r* = 0.992 (*p* < 0.001).

**Table 1:** Measurement of cSDH postoperative pneumocephalus volume by ABC/2 versus computer-assisted measurement

Overall postoperative pneumocephalus volume measurement	Mean	Minimum	Maximum
- ABC/2 method (ml)	16.26	0.20	123.40
- Computer-assisted measurement (ml)	18.04	1.11	128.45
Postoperative pneumocephalus volume measurement in *unilateral* burr-hole drainage for cSDH	Mean	Minimum	Maximum
- ABC/2 method (ml)	11.08	0.20	42.71
- Computer-assisted measurement (ml)	12.21	1.11	43.59
Total postoperative pneumocephalus volume measurement in *bilateral* burr-hole drainage for cSDH	Mean	Minimum	Maximum
- ABC/2 method (ml)	26.31	1.97	123.40
- Computer-assisted measurement (ml)	29.34	2.25	128.45

*cSDH* Chronic subdural haematoma, *ml* Millilitre

**Fig. 2:**
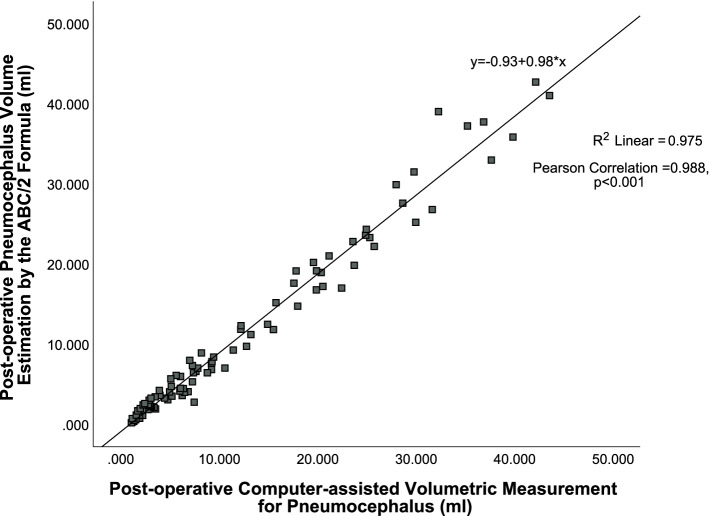
Excellent pneumocephalus volumes estimation by the ABC/2 method in postoperative cSDH after *unilateral* burr-hole drainage versus the gold standard by the computer-assisted volumetric measurement, *r* = 0.988 (*p* < 0.001)

There were 93 unilateral cSDH CT scans. The mean volume by computer-assisted measurement was 12.21 ml (range: 1.11 to 43.59 ml) versus 11.08 ml (range: 0.20 to 42.71 ml) by the ABC/2 method. The mean difference between the volume estimated by ABC/2 and the computer-assisted measurement was only −1.13 ml (range: −5.46 to 6.72 ml). The correlation coefficient was *r* = 0.988 (*p* < 0.001), as shown in Fig. 2.

**Fig. 3:**
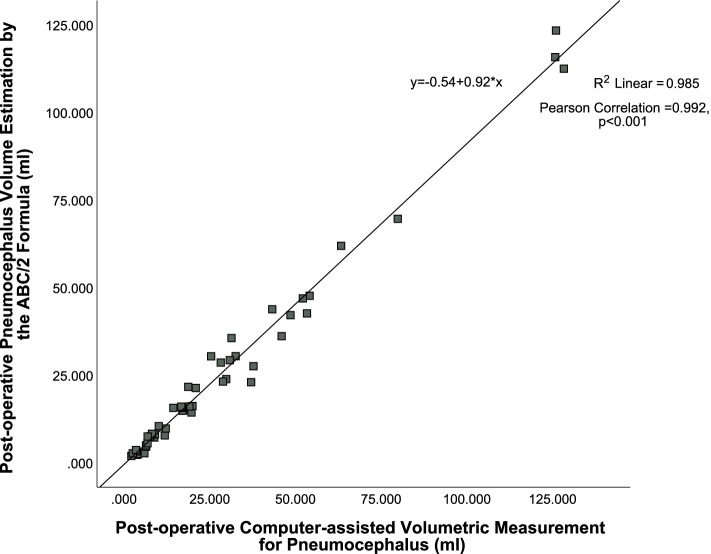
Excellent pneumocephalus volumes estimation by the ABC/2 method in postoperative cSDH after *bilateral* burr-hole drainage versus the gold standard by the computer-assisted volumetric measurement, *r* = 0.992 (*p* < 0.001)

**Fig. 4:**
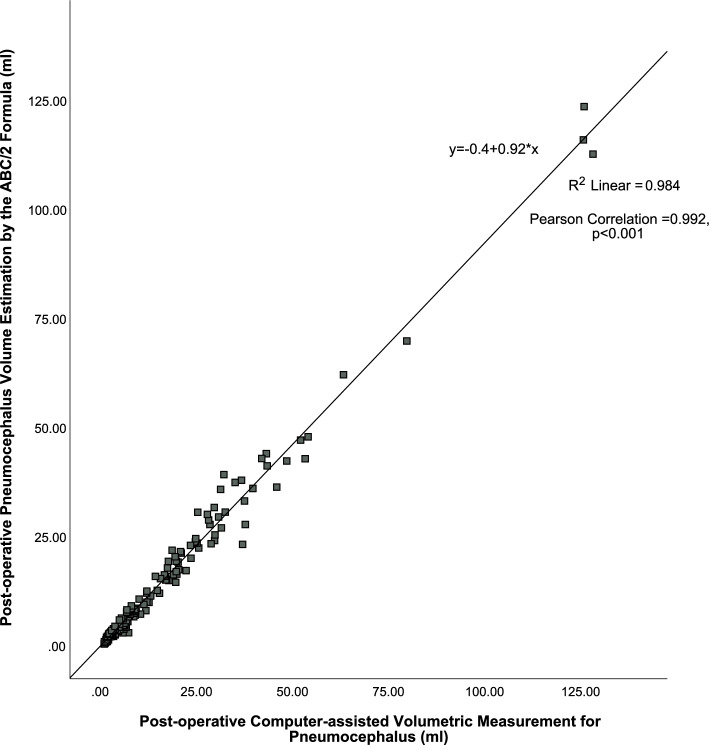
Excellent correlation in pneumocephalus volumes estimation by the ABC/2 method in *all* postoperative cSDH including both *unilateral* and *bilateral* burr-hole drainage versus the gold standard by the computer-assisted volumetric measurement, *r* = 0.992 (*p* < 0.001)

Forty-eight of the CT scans were bilateral cSDH. The mean pneumocephalus volume of bilateral cSDH by the computer-assisted measurement was 29.34 ml (range: 2.25 to 128.45 ml) versus 26.31 ml (range: 1.97 to 123.40 ml) by ABC/2, with *r* = 0.992 (*p* < 0.001), as shown in Fig. 3. The mean difference between the volume estimated by ABC/2 and the computer-assisted measurement was −3.03 ml (range: −15.95 to 4.89 ml). The postoperative pneumocephalus volume after bilateral burr-hole drainage was significantly larger than that of unilateral burr-hole drainage (29.34 ml versus 12.21 ml, *p* < 0.001). Overall, the mean difference between the volume estimated by ABC/2 and the computer-assisted measurement in all cases was only −1.78 ml (range: −15.95 to 6.72 ml). An excellent correlation between the ABC/2 and the gold standard was observed. The estimated volumes by the formula ABC/2 were significantly correlated to the volumes as measured by the computer-assisted volumetric technique in all postoperative pneumocephalus after unilateral and bilateral burr-hole drainage, with the correlation coefficient very close to 1, which signifies a very strong positive correlation (Fig. 4).

The overall recurrence rate of cSDH in this study was 6.06%. Among the 66 cases of cSDH with burr-hole surgery performed, 4 cases experienced a recurrence of chronic subdural hematoma requiring reoperation within 6 months. The mean interval between the 1st and 2nd operation for these four patients was 28 days (range: 16 to 38 days). The mean postoperation pneumocephalus volume of the 4 cases was 13.785 ml (range 3.626 to 22.807 ml).

## Discussion

To the best of our knowledge, there have not been studies specifically investigating the accuracy of this formula for pneumocephalus volume estimation in patients treated with burr-hole drainage for cSDH. The current study is one of the first with the largest number of pneumocephalus cases to evaluate the accuracy of the ABC/2 formula for postoperative pneumocephalus in chronic subdural hematoma (cSDH) patients after burr-hole drainage. An excellent correlation between the ABC/2 technique and computer-assisted measurement was achieved for pneumocephalus volume estimation in postoperative cSDH patients. The formula can be an easy tool to evaluate the volume of intracranial air at the bedside when clinicians are treating and monitoring the absorption of pneumocephalus. This simple verified formula for pneumocephalus volume estimation can be of value for daily clinical use and future large-scale clinical trials on cSDH. With the usage of normobaric oxygen therapy in treating pneumocephalus, the ABC/2 method can help researchers and clinicians evaluate the size of pneumocephalus and calculate the rate of air absorption [18, 19].

cSDH is a common condition encountered in the neurosurgical practice [20, 21]. Burr-hole drainage is an effective treatment for cSDH [22, 23]. However, the recurrence rate can be up to 5 to 33% [24–26]. Recurrence of cSDH in the elderly is associated with poor outcomes and mortalities [27, 28]. Pneumocephalus is common after surgical evacuation of cSDH [4]. The majority of the pneumocephalus would resolve spontaneously without major clinical complications [4]. However, in some patients with a significant amount of intracranial air, brain reexpansion may be hindered, which is in turn associated with the recurrence of cSDH [29]. Lutz et al. reported from a multivariate analysis of a randomized controlled trial (RCT) that limited intraoperative brain expansion (iBE) was a significant risk factor associated with recurrence [29]. You et al. reported that cSDH patients with more than 30 ml of postoperative pneumocephalus had a significantly higher recurrence rate [5]. Ohba et al. reported from a multivariate analysis that the presence of massive postoperative subdural air collection tended to be associated with recurrence [30]. Nakaguchi et al. reported that cSDH patients with residual subdural air as shown from the postoperative day 7 computed tomographic (CT) scan had a higher recurrence rate [31]. Amirjamshidi et al. reported that the presence of intracranial air 7 days after surgery was significantly associated with recurrence of cSDH [1]. It was associated with a longer length of stay in the hospital, recovery time, and functional outcome [5]. In severe cases, the development of tension pneumocephalus can be a life-threatening postoperative complication [32].

It is true that ABC/2 is a crude method for volume calculation, but it is the simplest one. It is derived from the formula πABC/6 for the volume calculation of elliptic spheres, so we can see that this formula is suitable for the calculation of lesions with structures similar to the elliptic spheres. Previous studies have found that the use of ABC/2 may overestimate the volume of AVMs. We believe the reason behind this was that most AVMs are conical or in other irregular shapes (other than an oval shape). ABC/2 is undoubtedly a more accurate measurement method for intracranial air accumulation with a shape more like an oval shape. This verified ‘bedside’ technique with ABC/2 can be a valuable tool in comparing volumes of postoperative intracranial air in cSDH patients after burr-hole drainage. This simple and reliable technique for pneumocephalus volume estimation can be of value for daily clinical application. It is also valuable to evaluate the treatment efficacy of various oxygen therapy for postoperative pneumocephalus [17, 33].

For the limitations of the study, it is retrospective in nature. Consecutive patients were analysed in order to minimize selection bias. One limitation is that the shape of the pneumocephalus might not be regular. Some smaller frontal pneumocephalus were oval in shape, while some larger pneumocephalus were crescent in shape. Another limitation of this study includes not analysing the accuracy of other possible formulas, such as ABC/3, Tada’s formula (p/ 6abc), or 2/3 Sh [34]. On the other hand, these other formulas such as ABC/3 were already shown to be less accurate in estimating ICH volumes [35]. Many studies, such as Gelal et al., had reported the XYZ/2 technique, or the ABC/2 formula can accurately estimate the volume of cSDH [7]. Hence, it is beyond the scope of our current paper to compare the accuracy of different mathematical formulas. Previous studies on the computer-assisted volumetric measurement of cSDH had reported limitations and inaccuracies. The CT images of cSDH were isodense or hypodense, and it was difficult for the software to accurately delineate the cSDH margins from the brain parenchyma based on Hounsfield units (HU). This ultimately led to some inaccuracy in the volume calculation by the “gold standard” computer-assisted method. On the other hand, for our current study, the intracranial air was well-delineated by the software based on the HU, and the volume was accurately calculated by the computer software as the gold standard for comparison.

## Conclusions

The ABC/2 method is an accurate and simple “bedside” technique to estimate the postoperative pneumocephalus volume. The estimated volume is highly correlated to the gold standard with computer-assisted volumetric measurement.

## Abbreviations

aSDH: Acute subdural haematoma
AVM: Arteriovenous malformation
cSDH: Chronic subdural haematoma
CT: Computed tomographic
EDH: Epidural haematoma
HU: Hounsfield units
ml: Millilitre
ICH: Intracranial haematoma
iBE: Intraoperative brain expansion
RCT: Randomized controlled trial
SRS: Stereotactic radiosurgery
SPSS: Statistical Package for the Social Sciences
